# First-generation protease inhibitor-triple therapy: SVR 24, safety, and predictors of response in a large single center cohort

**DOI:** 10.1186/s12985-015-0261-0

**Published:** 2015-03-03

**Authors:** Christoph R Werner, Carolin Franz, Daniel P Egetemeyr, Robert Beck, Nisar P Malek, Ulrich M Lauer, Christoph P Berg

**Affiliations:** Department of Gastroenterology, Hepatology, and Infectiology, University Hospital Tübingen, Tübingen, Germany; Institute of Medical Virology, University Hospital Tübingen, Tübingen, Germany

**Keywords:** Telaprevir, Boceprevir, Hepatitis C, Sustained virological response, Liver cirrhosis, Side effects

## Abstract

**Background/Aims:**

Aim of this retrospective study was to analyze the efficacy, safety, and predictors of treatment success for first-generation-PI triple therapies, including either boceprevir or telaprevir, in a mono-centric “real-life” setting with respect to SVR 24.

**Patients:**

131 patients (102 patients telaprevir, 29 patients boceprevir) were treated. Of these, 33/131 patients were treatment naïve, 72/131 patients had been pretreated with PEG-IFN/RBV (PR) (thereof: 36 with non-response, 30 with relapse, 6 unknown), and 26/131 patients previously had received non-pegylated interferon. 96/131 patients were infected with HCV genotype 1b. 41/131 patients had liver cirrhosis.

**Results:**

95/131 (73%) patients achieved SVR 24. SVR rates for subgroups were: 26/33 (79%) for treatment naïve, 25/30 (83%) for PR-relapse, 20/36 (56%) for PR-non-response, 21/26 (81%) for non-PR pretreated patients, (26/41) 63% for patients with liver cirrhosis, 23/35 (66%) genotype 1a, 72/96 (75%) genotype 1b. Predictors of SVR 24 were eRVR and a negative viral load at PI-treatment week 4 (p < 0.0001), negative predictors were quantifiable HCV viral load at PI-treatment week 4 (p < 0.0001), baseline platelet count < 100/nl (p < 0.0001), and previous PR-non-response (p = 0.006). 33/131 (25%) patients discontinued treatment prematurely, of those 14/131 (11%) patients due to virological failure. Side effects were frequent (anemia 59/131 [45%], severe infections 6/131 [5%]).

**Conclusions:**

According to our SVR 24 results, efficacy of PI-based triple therapy in our “real-life” cohort is comparable to the large multi-centric clinical trials. Pronounced side effects are frequent during therapy and often need complex therapeutic interventions. Since new DAA are available, it is open to discussion, if first-generation PI-triple therapy is no longer indicated at all.

## Introduction

The prevalence of hepatitis C virus (HCV) infection in western countries is in the range of 0.2-2%, while worldwide about 170 million people are threatened by the disease. In Europe and Northern America, HCV genotype 1 infections are most prevalent with figures of about 50% [1-3]. From the turn of the millennium until 2011 the standard of care for treatment of HCV consisted of pegylated interferon (PEG-IFN), and ribavirin (RBV), thus achieving sustained viral response (SVR) rates of about 50% for HCV genotype 1 patients [4-8]. Unfortunately, many HCV genotype 1 patients concluded the dual therapy without success and later on suffered from severe complications of advanced liver disease. Therefore, HCV genotype 1 infected patients were assigned as a “difficult-to-treat” group of patients, and a substantial fraction eventually had to undergo liver transplantation, or even finally deceased [9]. Therefore, as in HIV and HBV treatment, direct-acting antiviral agents (DAA) were desired. Several drug classes were in development: the (i) polymerase-, (ii) NS5A-, and (iii) protease inhibitors (PI). Finally, in 2011, first-generation PI boceprevir (BOC) and telaprevir (TVR) were approved by the authorities. PI-containing combinations constituted the treatment standard until early 2014 [10-14]. By adding BOC or TVR to the combination of PEG-IFN plus RBV (PR), SVR rates in treatment naïve patients were found to be significantly raised in clinical trials to up to 67-68% (BOC), and 75% (TVR) compared to a sole PR treatment exhibiting an SVR rate of only around 50% [4,5,10,13]. However, when compared to the hitherto PR standard regimen, the superb antiviral activity of these new triple therapy regimens was counteracted by aggravated side effects, namely anemia, bacterial infections, and dermatological toxicity.

Now, in the dusk of the era of first generation PI, with approval of (i) the first-in-class polymerase inhibitor Sofosbuvir in January of 2014, (ii) the second-generation protease inhibitor Simeprevir in May 2014 (in Europe, earlier in the US), and the approval of (iii) the NS5A inhibitors Daclatasvir and Ledipasvir later this year [15], we here now summarize our experiences with first-generation PI being obtained in an experienced tertial referral center. Aim of this retrospective analysis was (i) to gather data on the antiviral efficacy and safety of PI-based triple therapy with respect to SVR 24 weeks after conclusion of treatment (SVR 24), and (ii) to determine predictors of SVR 24, or premature discontinuation, respectively, in the “real-life” setting of a large single center cohort in the hands of an experienced treatment center.

### Patient characteristics

The clinical features of our study cohort are presented in Table 1. This retrospective analysis includes all 131 consecutively recruited patients, who were set on a PI-triple therapy including PR and TVR (102 patients), or PR and BOC (29 patients) between July 2011 and May 2012 at our center. From July 2011 until beginning of September 2011 all patients were treated with BOC-triple therapy, since TVR was not approved then. However, after approval of TVR in late September 2011, almost all patients were treated with the TVR-triple therapy at our center. This special management of patients explains the disproportion in numbers of both groups of patients.

**Table 1:** **Characteristics of study cohort patients**

**Demographics**		**TVR**	**BOC**
	n	102	29
Age	(years)*	53 (45.5-60)	50 (37.5-57.5)
Sex	Male/Female/total	53/49/102	20/9/29
Weight	(kg)*	77 (64–85)	78 (68.5-86)
BMI	(kg/m^2^)*	26 (23.1-28.7)	26 (24–28)
**Baseline viral characteristics**			
Genotype 1a/1b	n;%/n;%	28; 27%/74; 73%	7; 24%/22; 76%
Baseline viral load	(IU/ml)*	1.175 Mio (462,250-3.075 Mio)	1.02 Mio (503,000-4.005 Mio)
Baseline viral load ≤ 800.000 IU/ml	n;%	35; 34%	11; 38%
Baseline viral load ≥ 800.000 IU/ml	n;%	66; 65%	18; 62%
Baseline viral load missing	n;%	1; 1%	0
**Assessment of severity of liver disease**			
Liver histology available	n;%	60; 59%	17; 59%
Fibrosis score	Ishak*	4 (2–5)	4 (2–5)
Activity score	Ishak*	8 (6–9)	7 (3–8)
Cirrhosis (fibrosis score Ishak ≥ 5)	n;%	29; 28%	6; 21%
Clinical signs of advanced liver disease, but no histology**	n;%	5; 5%	1; 3%
Treatment history (last treatment***)			
Treatment naïve	n;%	24; 24%	9; 31%
PEG-IFN/RBV, overall	n;%	58; 57%	14; 48%
PEG-IFN/RBV, non-response^+^	n;%	28; 27%	8; 28%
PEG-IFN/RBV, relapse	n;%	25; 25%	5; 17%
PEG-IFN/RBV, unknown response	n;%	5; 5%	1; 3%
Non-PEG IFN overall^++^	n;%	20; 20%	6; 21%
Non-PEG IFN ± RBV, non-response	n;%	9; 9%	4; 4%
Non-PEG IFN ± RBV, relapse	n;%	9; 9%	2; 2%
Non-PEG IFN ± RBV, unknown response	n;%	2; 2%	0
**Current treatment characteristics**			
PEG-IFN 2a/2b	n;%/n;%	96; 94%/6; 6%	27; 93%/2; 7%
RBV baseline dosage	mg/day*	1200 (1000–1200)	1200 (1000–1200)
RBV baseline dosage per body weight	mg/kg body weight/day*	14.6 (14.1-15.65)	15 ± 2.6 (14.4-15.9)
**Baseline clinical chemistry**			
Leukocytes	(/μl)*	5990 (4842–7295)	6645 (5330–8410)
Hemoglobin	(g/dl)*	14.7 (13.8-15.9)	15.3 (14.4-15.9)
Platelets	(thousand/μl)*	186 (143–257)	227 (182–259)
Creatinine	(mg/dl)*	0.7 (0.6-0.8)	0.7 (0.6-0.8)
GFR MDRD	(ml/minute)*	102 (90–119)	112.8 (101.6-126.4)
Total Bilirubin	(mg/dl)*	0.7 (0.6-0.9)	0.7 (0.5-0.9)
Quick	(%)*	105 (95–112)	108 (98–115)
INR	INR*	1 (0.9-1)	1 (0.9-1)
GPT	IU/l*	69 (44–87)	64 (46–97)

*Data are presented as medians (interquartile ranges in parentheses); **e.g. esophageal varices, ascites, distinct sonographical signs of portal hypertension or liver cirrhosis; ***treatment with highest antiviral activity, if low dose PEG-IFN, then neglected; ^+^including “null-responders”, “partial responders”, and one patient with “viral breakthrough”; ^++^including interferon alpha 2a, interferon alpha 2b, and consensus interferon. Abbreviations: *BMI* Body mass index, *GFR MDRD* Gomerular filtration rate modification of diet in renal disease, *GPT* Glutamate-pyruvate transaminase, *INR* International normalized ratio, *PEG-IFN* Pegylated interferon, *RBV* Ribavirin.

The treatment course for TVR-based triple therapy consisted of 12 weeks of triple therapy with TVR and PR, followed by a dual therapy with PR. The length of that second dual therapy period was variable, according to the approved treatment recommendations: if patients were (i) treatment naïve or relapsers to a classical dual treatment regimen consisting of PR alone, (ii) had no liver fibrosis of higher grade, and (iii) exhibited a negative HCV viral load (Cobas AmpliPrep/Cobas TaqMan HCV Test, Roche Diagnostics GmbH, Mannheim, Germany; lower limit of detection [LLOD] and lower limit of quantification [LLOQ] 15 IU/ml,) after 4 weeks of triple therapy, they were eligible for treatment shortening down to altogether 24 weeks; if not so, treatment was extended to 48 weeks. According to guidelines, those patients with non-pegylated IFN as last treatment were eligible for treatment shortening as well, even if they were classified as non-responders as last treatment response. Patients, who presented with a HCV viral load above 1000 IU/ml at treatment week (TW) 4 or at any time afterwards had to discontinue treatment prematurely; the same applied for patients, who exhibited a rise in HCV viral load of 1 log_10_ above a previous nadir viral load.

BOC-based triple therapy was conducted in accordance with the European and German regulations (which differ from the treatment schedule in the US): following a “lead-in” phase of four weeks of sole PR treatment, BOC was added to the dual treatment regimen. Duration of triple therapy differed according to the respective fibrosis grade and previous treatment responses: if patients were treatment naïve and achieved a HCV RNA level below LLOD after TW 8, treatment duration could be shortened to 28 weeks altogether. When a patient was treatment naïve, and failed to achieve a HCV RNA level below LLOD at TW 8, but reached this goal thereafter (until TW 24) and additionally had no proven liver cirrhosis, then BOC could be abandoned after TW 36, followed by additional 12 weeks with PR alone until TW 48. If the patient had been on PR previously (resulting in virological relapse, breakthrough, or confirmed “partial response”), demonstrated a HCV viral load below LLOD after TW 24, and additionally had no proven liver cirrhosis, then BOC could be abandoned after TW 36, followed by additional 12 weeks with PR alone until TW 48. In patients with liver cirrhosis and/or previous non-response (null-response) to a PR treatment, BOC had to be given in addition to PR from TW 4 on to TW 48. Patients, who presented with a HCV viral load above 100 IU/ml at TW 12 or at any time afterwards had to discontinue treatment prematurely; the same applied for patients, who exhibited a rise in HCV viral load of one log_10_ above a previous nadir viral load. Due to our status as a tertiary referral center (being quite used to referrals of cases exhibiting incomplete data on viral loads in previous treatment attempts performed “outside”), we *a priori* did not differentiate between so called “null-response” and “partial response” patients, and subsumed those patients as “non-response” patients, a procedure which was commonly accepted in the pre-PI era.

In response to anemia, we administered EPO based on individual decisions as follows: (i) if patients were symptomatic or (ii) if we assumed that RBV dose reduction could be avoided or (iii) if we assumed that we would have to reduce RBV dosing to a lesser extent when applying EPO. Granulocyte-colony stimulating factor (G-CSF) was given if patients exhibited leukocyte counts below 1000/μL.

Baseline laboratory values are shown in Table 1. 4/131 patients presented with leukopenia (leukocyte count < 2500/μl), 15/131 patients had a moderate (platelets < 100/nl) and 2/131 a severe thrombopenia (platelets < 50/nl). All of them, except 3 patients with moderate thrombopenia, were in the TVR patient group. One of those patients had immune-mediated thrombopenia. Transaminases were found to be elevated in 113/131 patients (GPT > 35 IU/ml: TVR-group 87/102; BOC-group 26/29, respectively). 3/131 patients exhibited a Quick value below 60% (one of those was on phenprocoumon for atrial fibrillation); all of them were in the TVR subset of patients. Baseline and follow-up data of albumin levels were available only on an occasional basis. Data were statistically analyzed using Microsoft Office Excel, Graph Pad Prism 6.0, and SPSS 21.

## Results

### Subgroup analysis of sustained virological response (SVR 24) rates

**Figure 1:**
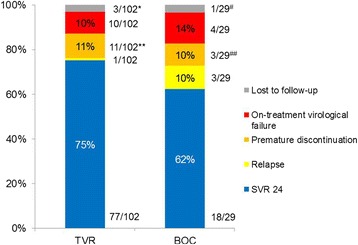
**Overall treatment outcome in the TVR and BOC patient subgroups (ITT).** *two of those patients reached SVR 12, but did not show up to SVR 24; **four patients reached SVR 24 despite premature discontinuation and therefore were added to the SVR 24-column; ^#^one patient lost to follow-up; ^##^one patient reached SVR 24 despite premature discontinuation and therefore was added to the SVR 24-column. Abbreviations: BOC: boceprevir, TVR: telaprevir; SVR: sustained virological response.

**Figure 2:**
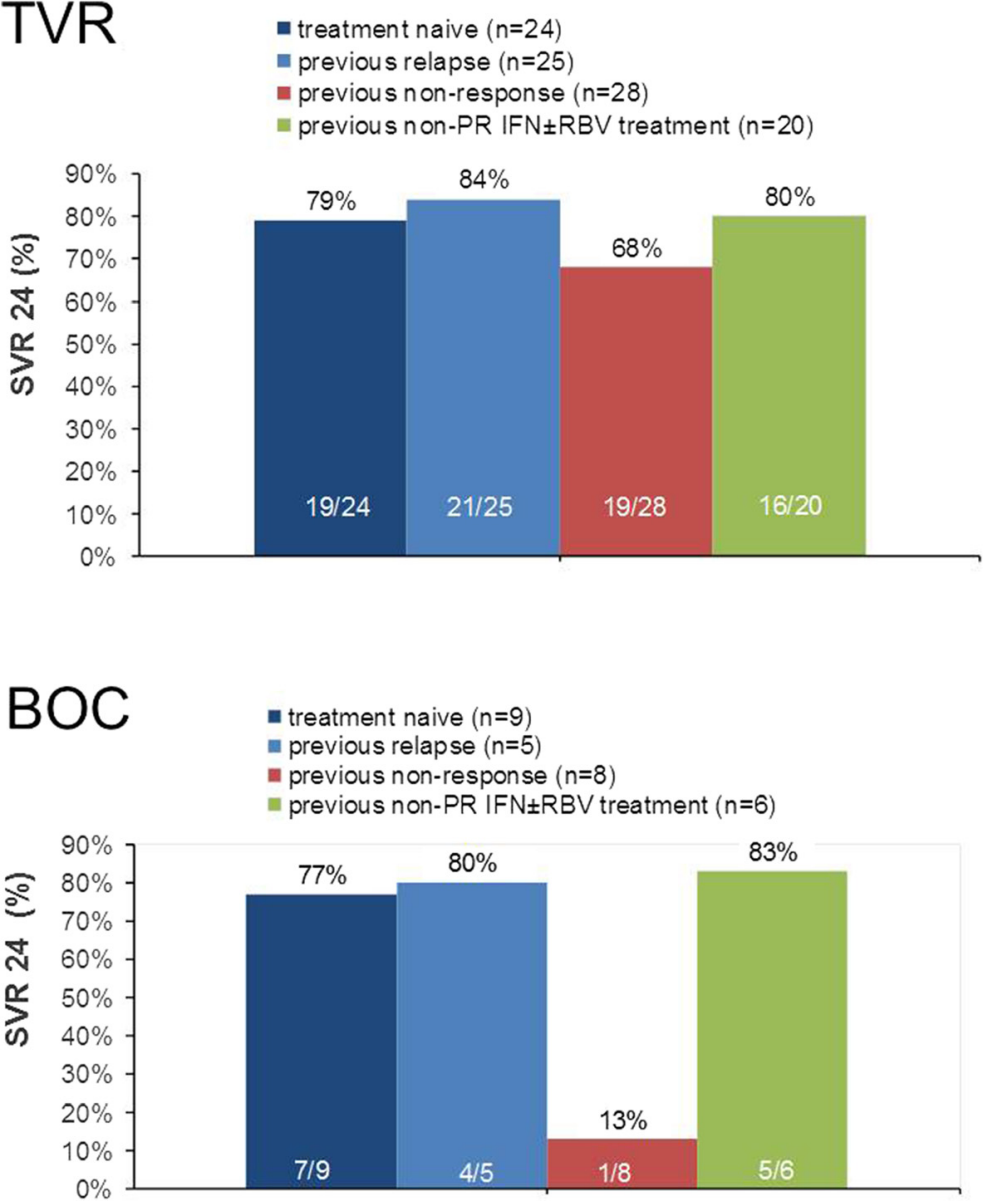
**SVR 24 rates with respect to previous treatment response (ITT).** Abbreviations: BOC: boceprevir, IFN: interferon, TVR: telaprevir; PR: pegylated interferon/ribavirin, RBV: ribavirin, SVR: sustained virological response. Missing patients had an unknown treatment outcome in a previous treatment.

Overall viral response rates and SVR 24 rates according to previous treatment response (naïve, relapse, non-response, non-pegylated IFN pretreated) are shown in Figures 1 and 2. In the overall cohort, 73% (95/131) of patients achieved SVR 24. Four patients were lost to follow-up. Of those four patients, 2 had reached SVR 12, but then did not show up for follow-up visits. SVR 24 rates in patients, (i) who were treatment naïve, (ii) suffered from virological relapse after a PR treatment, or (iii) had a non-PEG-IFN-based previous treatment, ranged from 77-84%, irrespective of the administered type of PI-triple therapy.

### Predictors of SVR 24

For evaluation of predictors of SVR 24, see Tables 2 and 3. By univariate analysis of the overall cohort, extended rapid virological response (eRVR; p < 0.0001) and a negative viral load at PI-TW 4 (p < 0.0001) were significantly associated with an increased likelihood of SVR 24, while a baseline platelet count of < 100/nl (p < 0.0001), a viral load above LLOQ at TW 4 (p < 0.0001), and a previous non-response to PR therapy (p = 0.006) were negative predictors of SVR 24. In the TVR-group of patients, the best positive predictor for SVR 24 was an eRVR, being defined as negative HCV-viral loads (below LLOD) at both TW 4, and 12, respectively. In our TVR-cohort, 64 out of 102 (63%) patients met those criteria. When TVR-treated patients achieved eRVR, the probability of achieving a SVR 24 later on was 91% (58/64 patients, p < 0.0001, Fisher’s exact test). For BOC triple therapy, due to the different treatment schedule, eRVR is defined as negative HCV-viral loads at both TW 8, and 24, respectively. In our BOC-cohort, patients, who achieved eRVR had a probability of 81% (13/16 patients) of achieving a SVR 24.

**Table 2:** **Predictive model of SVR analyzed for all patients treated with PI-triple therapy (TVR + BOC; n = 131), and analyzed for subgroups of patients treated with TVR (n = 102) or BOC (n = 29)**

	**SVR 24**	**p value**	**SVR 24**	**p value**	**SVR 24**	**p value**
	**(TVR + BOC)**	**(TVR + BOC)**	**(TVR)**		**(BOC)**	**(BOC)**
	**(n/N;%** ^**#**^ **)**		**(n/N;%** ^**#**^ **)**	**(TVR)**	**(n/N;%** ^**#**^ **)**	
**Viral kinetics** ^**##**^						
eRVR	71/80; 89%	**<0.0001**	58/64; 91%	**<0.0001**	13/16; 81%	0.0969
Non-eRVR	23/45; 51%		18/34; 53%		5/11; 45%	
PI-TW 4 < LLOD	72/83; 87%	**<0.0001**	59/67; 88%	**0.0005**	13/16; 81%	0.0969
PI-TW 4 > LLOD	22/42; 52%		17/31; 55%		5/11; 45%	
PI-TW 4 < LLOD	72/83; 87%	**0.0193**	59/67; 88%	0.0619	13/16; 81%	0.5528
PI-TW 4 minimal viral load (> LLOD, < LLOQ)	20/29; 69%		17/24; 71%		3/5; 60%	
PI-TW 4 viral load < LLOQ	92/112; 82%	**<0.0001**	76/91; 84%	**<0.0001**	16/21; 76%	0.1358
PI-TW 4 viral load > LLOQ	2/13; 15%		0/7; 0%		2/6; 33%	
**Baseline demographic parameters**						
**Fibrosis**						
Liver Cirrhosis (Ishak 5 + 6)	26/41; 63%	0.0931	24/34; 71%	0.3331	2/7; 29%	0.0712
No Liver Cirrhosis (Ishak 1–4)	70/90; 78%		54/68; 79%		16/22; 73%	
**Sex**						
Male	49/73; 67%	0.1675	39/53; 74%	0.6541	10/20; 50%	0.0959
Female	46/58; 79%		38/49; 78%		8/9; 89%	
**Age**						
Patients ≥ 60 years	23/31; 74%	1.0000	19/26; 73%	0.6053	4/5; 80%	0.6221
Patients < 60 years	73/100; 73%		59/76; 78%		14/24; 58%	
**Baseline viral load** ^**###**^						
High viral load (>800.000 IU/ml)	60/84; 71%	0.6804	49/66; 74%	0.8126	11/18; 56%	0.6942
Low viral load (<800.000 IU/ml)	35/46; 76%		27/35; 77%		8/11; 73%	
**Genotype**						
1a	23/35; 66%	0.3763	19/28; 68%	0.3068	4/7; 57%	1
1b	72/96; 75%		58/74; 78%		14/22; 64%	
**Baseline platelet count**						
Platelets > 100/nl	91/116; 78%	**0.0001**	73/90; 81%	**0.0012**	18/26; 69%	**0.0452**
Platelets < 100/nl	4/11; 36%		4/12; 33%		0/3; 0%	
Previous non-response vs. other*						
Non-response	20/36; 55%	**0.0075**	19/28; 68%	0.2948	1/8; 13%	**0.0014**
Other	76/95; 80%		59/74; 80%		17/21; 81%	

Fisher’s exact test was used. Significant calculations (p < 0.05) are printed bold. ^#^number of patients who achieved SVR 24 in category/total number in category. ^##^6 patients (4 TVR-, 2 BOC-patients) had no measurement of viral load at TW 4 due to premature treatment discontinuation or because they did not show up for scheduled visit; therefore they were excluded from analysis. ^###^1 baseline viral load missing. *Other (includes treatment naïve, relapsers, unknown response, pretreatment with other interferon than pegylated interferon). Abbreviations: *BOC* Boceprevir, *eRVR* Extended rapid virological response, *LLOD* Lower limit of detection, *LLOQ* Lower limit of quantification, *PI* Protease inhibitor, *SVR* Sustained virological response, *TVR* Telaprevir, *TW* Treatment week.

**Table 3:** **Univariate and multivariate models for prediction of SVR (for all patients treated with PI-triple therapy; n = 131)**

	**Univariate analysis**		Multivariate analyses**	
	**Odds ratio (95% CI)**	**Wald p value**	**Odds ratio (95% CI)**	**Wald p value**
**Viral kinetics**				
eRVR vs. no eRVR	**8.875 (3.66, 21.51)**	**<0.0001**	**8.875 (3.66, 21.51)**	**<0.0001**
PI-TW 4 < LLOD vs. > LLOD	**7.115 (3.04, 16.65)**	**<0.0001**		n/a***
PI-TW 4 viral load > LLOQ vs. < LLOQ	**0.049 (0.01, 0.24)**	**<0.0001**		n/a***
**Baseline demographic parameters**				
**Fibrosis**				
Liver Cirrhosis (Ishak 5 + 6) vs. no Liver Cirrhosis (Ishak 1–4)	0.528 (0.24, 1.18)	0.118		0.858
**Sex**				
Male vs. female	0.533 (0.239, 1.187)	0.123		0.194
**Age**				
Patients < 60 years vs. ≥ 60 years	0.894 (0.358, 2.23)	0.811		0.751
**Baseline viral load**				
High viral load (>800.000 IU/ml) vs. Low viral load (<800.000 IU/ml)	0.679 (0.29, 1.58)	0.367		0.165
**Genotype**				
1a vs. 1b	1.565 (0.678, 3.62)	0.294		0.308
**Baseline platelet count**				
Platelets < 100/nl vs. > 100/nl	**0.1 (0.029, 0.341)**	**<0.0001**	**0.112 (0.032, 0.394)**	**0.001**
**Previous treatment response**				
Non-response vs. other*	**0.313 (0.137, 0.715)**	**0.006**	**0.357 (0.147, 0.867)**	**0.023**

Significant calculations (p < 0.05) are printed bold. For absolute numbers see Table 2. *Other (includes treatment naïve, relapsers, unknown response, pretreatment with other interferon than pegylated interferon). **Multivariate analysis was performed twice: first, all shown parameters were included, showing significant results for “eRVR vs. no eRVR”, and “Platelets < 100/nl vs. > 100/nl”, secondly multivariate analysis was conducted only with “baseline demographic parameters” (excluding parameters of viral kinetics), showing significant results for “Platelets < 100/nl vs. > 100/nl”, and additionally “Non-response vs. other”. ***“PI-TW4 < LLOD vs. > LLOD” and “PI-TW 4 viral load > LLOQ vs. < LLOQ” both had to be excluded from the multivariate analysis due to strong collinearity with “eRVR vs. no eRVR”. The width of the confidence intervals might be due to the limited number of cases in our sample. Abbreviations: *eRVR* Extended rapid virological response, *LLOD* Lower limit of detection, *LLOQ* Lower limit of quantification, *PI* Protease inhibitor, *SVR* Sustained virological response, *TW* Treatment week, *n/a* Not applicable.

For the overall cohort, the probability of achieving a SVR 24 was lower in patients, who had a detectable, but not quantifiable viral load (below LLOQ) at TW 4: In this subset of patients, only 69% (20/29 patients) achieved a SVR 24 (p = 0.0193, Fisher’s exact test). Reasons for treatment failure were virological failure (n = 4), premature discontinuation (n = 4), and relapse (n = 1).

At least in our cohort, for TVR-patients the following prediction could be made: if patients showed a quantifiable viral load 4 weeks after onset of PI-administration (viral load above LLOQ at TW 4), no patient (0/7 patients) achieved a SVR 24 later on, in all cases due to virological failure (p < 0.0001, Fisher’s exact test).

15 out of 131 patients (12 patients TVR-treated, 3 patients BOC-treated, respectively) had platelet counts below 100/nL at baseline thus resembling an extra-“difficult-to-treat” group of patients [16]. Of those, just two patients achieved SVR 24 after regular completion of a full treatment course (13%), and thus had a significantly lower SVR 24 rate than others (p = 0.0001, Fisher’s exact test).

Patients who previously had shown a virological non-response to PR treatment, exhibited lower SVR 24 rates than other subgroups (p = 0.0075), especially in the BOC group of patients (p = 0.0014, Fisher’s exact test).

Almost all patients with a virological failure during PI-triple therapy (13/14 patients) had a high viral load (> 800.000 IU HCV RNA/ml) at baseline. However, no statistical significance was achieved with respect to predictability of SVR 24, at least in our cohort. Older patients (> 60 years), knowingly prone to side effects, are “difficult-to-treat” patients [17]. However, in our cohort, irrespective of previous treatment status, we could achieve favorable SVR rates in this subgroup of patients (73% with TVR, and 4 out of 5 patients with BOC, respectively).

In the TVR-subset, patients with liver cirrhosis achieved SVR 24 rates of 71%, and remarkably, in the subgroup of TVR-patients, who had both liver cirrhosis and were of older age, a SVR 24 rate of 67% (8/12 patients) could be achieved. In the BOC group of patients with liver cirrhosis 2 out of 7 patients achieved SVR 24.

Multivariate analysis identified eRVR, a baseline platelet count of > 100/nl, and previous response status as independent predictors of higher SVR 24 rates (see Table 3 for odds ratios, and confidence intervals).

### Response guided therapy

In the TVR-group at baseline 48 of the 102 patients had no fundamental contraindications for a shortened treatment course of 24 weeks, as e.g. liver cirrhosis or previous non-response to a PR therapy. Of those, 34 out of 48 (71%) were found to reach a HCV viral load below LLOD at TW 4, thus meeting the basic requirement for treatment shortening. In fact, treatment shortening finally was implemented in only 26 out of 48 patients (54%). 25 out of those 26 patients (96%) achieved SVR 24, whereas 1 patient was lost to follow-up. With BOC-triple therapy, 4 out of 7 patients, who had no basic contraindications for shortening of treatment at baseline, met all criteria at TW 8 for an implementation of such a procedure. Of those, two patients achieved SVR 24, while one patient suffered from virological relapse and another patient was lost to follow-up.

### Virological failure

Altogether, 14 of the 131 patients (11%) had to stop PI-based triple therapy due to on-treatment virological failure (see Figure 1). Additionally, 4 patients (3%) suffered from virological relapse during follow-up, all of those occurred between EOT and first follow-up, which was routinely done 12 weeks after EOT. 10 of the 102 TVR-patients (10%) suffered from on-treatment virological failure (4 patients meeting the futility rule of a viral load > 1000 IU/ml, 3 patients with virological breakthrough at TW 12, 18, and 24, respectively, and 3 patients with rising viral loads after having reached a nadir). Additionally, 1 patient experienced a virological relapse. In the TVR-group of patients no virological failure occurred after TW 24. In the BOC-group of patients, altogether 4 out of 29 patients (14%) experienced an on-treatment virological failure: 3 patients suffered from virological non-response, when meeting the futility rule (HCV viral load > 100 IU/ml) at TW 12 or later. One patient suffered from virological breakthrough at TW 48. Additionally, 3 patients (10%) experienced a virological relapse between EOT and first follow-up visit.

### Discontinuation of treatment

Altogether, 33 out of 131 (25%) patients prematurely discontinued PI-triple therapy. Of those, 14 discontinued due to virological failure (see above) and 18 due to side effects. One additional patient belonging to the TVR-patient cohort was treated after having been listed for liver transplantation due to hepatocellular carcinoma. This patient qualified for an exceptional MELD score before and during therapy. At TW 36 an organ offer by EUROTRANSPLANT had been accepted for this patient followed by successful transplantation. This patient therefore discontinued treatment prematurely. Nevertheless, this patient stayed HCV negative thereafter in a sustained fashion (SVR 24). In the TVR-group of patients, 14 other patients prematurely discontinued complete treatment: 4 patients due to rash, 3 patients due to infections, 3 patients due to intolerance of the treatment, 2 patients due to hepatic decompensation, 1 patient due to intracerebral bleeding, and 1 patient due to lung cancer. Despite their premature discontinuation of treatment, three of those 14 patients achieved a SVR 24, including one patient, who had to undergo LTx due to hepatic decompensation at TW 4.

In the BOC-group of patients, 4 patients discontinued treatment prematurely: Two patients due to intolerance to the treatment, one patient due to non-treatment associated posttraumatic stress syndrome, and one patient due to pruritus. The latter one nevertheless achieved a SVR 24.

Remarkably, 14 out of the 18 discontinuations which had been due to side effects had occurred in patients of older age (≥ 60 years), or in patients with liver cirrhosis, among those 5 patients exhibiting a liver cirrhosis at age ≥ 60 years. This again emphasizes the “difficult-to-treat” status of these subgroups.

In 4 of the 102 TVR-treated patients TVR alone was withdrawn prematurely due to development of a severe rash. Additionally, one of the 29 BOC-treated patients discontinued taking BOC due to diarrhea. Subsequently, PR treatment was continued in all patients. All five patients achieved SVR 24 later on.

### Side effects

Side effects were frequent during PI-triple therapy, and so were therapeutic interventions. However, no patient was lost during therapy.

Hematological toxicity is a specific feature of PI-triple therapy, irrespective of which PI has been used (see Table 4 for details). Thus, therapeutic interventions were frequent and had to be performed in 36% (TVR), and 28% (BOC), respectively. The difference between the high rate of anemia below 10 g/dL (45%), and the lower rate of RBV reduction is most probably due to our policy, not to reduce RBV dosage in asymptomatic or oligosymptomatic patients with anemia with hemoglobin values above 8.5 g/dL during the first phase of PI-triple therapy. When at the EASL 2012 preliminary results from the study of Poordad et al. [18] were published, showing no difference in outcome if the RBV dosage is significantly reduced, we changed our policy, and reduced RBV dosage at an earlier stage, contrary to the treatment with PEG-IFN and RBV alone, where RBV reduction if ever possible should be avoided. Nevertheless, no patient had to discontinue treatment due to anemia.

**Table 4:** **Hematological side effects in our “real-life” cohort**

**Hematological side effects: Leukocytes**		**TVR**	**BOC**
Leukocytes < 2500/μl	n/N;%	68/102; 67%	18 29; 62%
Leukocytes < 1500/μl	n/N;%	18/102; 18%	2/29; 7%
**Hemoglobin**			
Hemoglobin < 10 g/dl	n/N;%	47/102; 46%	12/29; 41%
Hemoglobin < 8.5 g/dl	n/N;%	21/102; 21%	5/29; 17%
Average decrease in hemoglobin from BL	g/dl	4.6 ± 1.7	4.8 ± 1.7
**Platelets**			
Platelets < 50/nl	n/N;%	18/102; 18%	0/29
Platelets < 20/nl	n/N;%	3/102; 4%	0/29
**Therapeutic interventions due to hematological side effects**			
RBV dose reduction	n/N;%	37/102; 36%	8/29; 28%
Combination of erythropoetin administration and blood transfusion*	n/N;%	20/102; 20%	5/29; 17%
Erythropoetin administration alone	n/N;%	9/102; 9%	2/29; 7%
Blood transfusion alone*	n/N;%	5/102; 5%	0/29
PEG-IFN dose reduction	n/N;%	7/102; 7%**	1/29; 3%
G-CSF administration	n/N;%	7/102; 7%	0/29

*Blood transfusion encompassing at least 2 units of concentrated erythrocytes; **due to leukopenia (n = 5), due to thrombocytopenia (n = 2). *BL* Base line.

Severe rash was a rare event in former dual treatment PR regime, and mostly was attributed to RBV. In PI-triple therapy, pruritus and rash seem to constitute a PI class effect, but in frequency and intensity much more associated with TVR than with BOC: 11 of the TVR-treated patients developed a severe rash, resulting in the necessity of a complete discontinuation of treatment in 4 patients, and premature discontinuation of TVR alone in additional 4 patients.

During PI-triple therapy, hospital admissions were required in 23 patients (21 TVR-treated, 2 BOC-treated) with some of those patients being hospitalized more than once. Hospitalizations were due to infections (6 patients), rash (5 patients), hepatic decompensation (4 patients), and anemia (2 patients), respectively. Further hospital admissions occurred due to a non-ST elevated myocardial infarction (hemoglobin level in this patient at that time point 12.9 g/dl), severe headaches, exsiccosis/ diarrhea, deep venous thrombosis, diagnosis and treatment of lung cancer, and performance of LTx, respectively.

During follow-up, four patients developed hepatocellular carcinoma. All of those patients had discontinued the antiviral treatment prematurely due to side effects (n = 3) or virological failure (n = 1) before the diagnosis of HCC was made. Of those, one patient underwent LTx later on. One patient was diagnosed with a malignant brain tumor one year after successful conclusion of BOC-triple therapy, and one patient died from an accident during follow-up.

## Discussion

Our single center retrospective study analyzing efficacy, safety and predictors of SVR 24 of PI-triple therapy in a “real-life” setting shows overall SVR rates similar to those, which had been achieved in the large clinical trials leading to the approval of both TVR and BOC for treatment of HCV, genotype 1, in 2011 [10-14,19,20]. Due to the retrospective character of our study and the relatively small number of patients, a comparison to the large prospective clinical trials is only of limited significance. Furthermore, due to the disproportion between both subsets of patients being either treated with BOC or TVR, respectively, no comparison between both groups is possible. However, this disproportion in distribution of both PI is present in other “real-life” cohorts [21].

In PI-triple therapy, eRVR was the best predictor for treatment success (SVR 24) both in our cohort and in the large clinical trials. Patients, who displayed a minimal viral load (> LLOD, < LLOQ) at PI-TW 4 (TVR: TW 4, BOC: TW 8) later on had a lower SVR rate compared with patients, who had no detectable viral load at this time-point (p = 0.0193). Thus, the achievement of a *negative* HCV viral load at PI-TW 4 is crucial for assessment of eRVR and further treatment decisions, including response guided treatment. Furthermore, at least in our cohort, TVR-patients, who showed a quantifiable viral load at PI-TW4 (> LLOQ), always failed to achieve SVR 24 at least in our cohort (p < 0.0001).

With respect to several subgroups of patients according to previous treatment, we found SVR rates in the range of the large clinical trials. Interestingly, our group of patients being previously treated with non-PEG-IFN exhibited the same SVR 24 rate as our cohorts of treatment naïve patients (80% [TVR], and 83% [BOC], respectively). However, since there are no large numbers of patients, and no controlled trials addressing non-pegylated interferon experienced patients, treatment decisions as shortening of treatment should be implemented with caution in this subgroup, at least in patients with a previous non-response to non-pegylated interferon.

Non-response to a previous PR treatment still is a negative predictor of treatment success in the era of first generation PI (p = 0.0075), although in our TVR-subgroup of patients we could achieve a SVR rate of 68%, thus exceeding the SVR rates noticed in the REALIZE trial [12]. In the large BOC-trials, “null-response” patients were omitted. However, in the different treatment arms of the RESPOND-2 trial, SVR rates of 40-52% could be achieved for the “partial response” patients [14]. In our group of merged non-response patients, just one out of 8 patients achieved the SVR status (13%). As a matter of fact, the small number of patients again is a severe limitation to this evidence.

Almost all patients with a virological failure during PI-triple therapy (13/14 patients) had a high viral load (> 800.000 IU HCV RNA/ml) at baseline. However, no statistical significance was achieved with respect to predictability of SVR 24, at least in our cohort.

Similar to the former standard treatment with PR, liver cirrhosis was found to constitute a negative predictor of SVR 24 also in PI-triple therapy [22], even though, at least in our TVR cohort, far better SVR rates could be achieved in comparison to former PR treatment. Our SVR 24 results varied between the two different PI-triple therapy groups of patients: In the TVR group of patients we could achieve SVR 24 rates of 71%, with side effects being the main limiting factor in this cohort, leading in 25% of patients to treatment discontinuation. These data exceed SVR 12 results from the French CUPIC cohort, with decreased SVR 12 rates in comparison to other subgroups of patients (SVR 12 in 132 out of 299 patients, or 44%; [16]), and other cohorts [22]. However, due to the small number of patients in comparison to these trials, these results should be interpreted with caution. With BOC-triple therapy, the CUPIC cohort observed a SVR 12 of 38% (80/212; [16]), while in our small cohort of patients with liver cirrhosis, merely two out of seven patients achieved SVR. Importantly, patients with significant portal hypertension defined by a baseline low platelet count (below 100/nl) seem not to be suitable for a first-generation PI-triple therapy, since almost all of those patients failed to reach EOT (11 out of 15 patients), either due to virological failure, or due to mostly severe side effects. This finding could also be observed in the CUPIC study [23]. Therefore, an exclusion of those patients from first-generation PI triple therapy is a reasonable treatment decision due to the disproportion between efficacy and potential harm of those treatment regimes.

In our cohort, older patients achieved good SVR rates (73% with TVR, and 80% with BOC, respectively). However, in aged patients, treatment failure reflects more the intolerance to side-effects than the lack of virological response to PI-triple therapy.

Of note, approximately half of treatment failures were due to side-effect induced premature discontinuations. Most of them occurred in the difficult-to-treat subgroups of cirrhotic patients or/and patients of older age (14 out of 18 patients, who discontinued due to side effects). While in the TVR-group of patients premature discontinuations of treatment due to severe side effects (rash, hepatic decompensation, infections, bleeding) occurred regularly, such intense side effects in general were not seen in the BOC-group of patients, but, however, in both groups the rates of discontinuations were around 10%.

Unlike to the large clinical trials, in our TVR-“real-life” cohort 5% (6/131) of patients had to be hospitalized due to bacterial infections. Other “real-life” cohorts reported severe infections in 2-9% of patients, with most severe infections in the group of patients with liver cirrhosis [16,21,24].

Even if TVR and BOC both still are mentioned in the current EASL treatment recommendations as of April 2014 with a half-sentence [25], in western countries the time for first-generation PI-treatment seems to be over. With the approval of Sofosbuvir, Dasabuvir, Simeprevir, Paritaprevir, Daclatasvir, Ledipasvir, and Ombitasvir [26-30], Interferon-free combinations are or will be available with SVR rates exceeding 90% through all genotypes. Importantly, these treatment regimens will have a by far more favorable safety profile than the first-generation PI-triple therapy with BOC and TVR.

However, since the new generation DAA possibly will not be available or affordable in all countries, TVR and BOC may find their niche in the treatment of certain subgroups of patients, or regions of the world.

## Conclusion

In summary, in our retrospective analysis SVR 24 rates of PI-triple therapy from the large prospective clinical trials could be translated into “real-life”. As in the large trials, eRVR is the strongest predictor of treatment success. Furthermore, not to achieve a HCV viral load below LLOQ at PI-TW 4 is a strong predictor of future treatment failure. Pronounced side effects are frequent during therapy and often need complex therapeutic interventions. Importantly, patients with advanced portal hypertension (platelets below 100/nl) should not be treated with PI-triple therapy due to low efficacy and an unfavorable safety profile.

## Abbreviations

CUPIC: Compassionate use of protease inhibitors in cirrhotics
DAA: Direct-acting antiviral agents
EOT: End of treatment
eRVR: Extended rapid virological response
G-CSF: Granulocyte-colony stimulating factor
GPT: Glutamate-pyruvate transferase
HCC: Hepatocellular carcinoma
HCV: Hepatitis C virus
IFN: Interferon
ITT: Intention to treat
IU: International units
LLOD: Lower limit of detection
LLOQ: Lower limit of quantification
LTx: Liver transplantation
MELD: Model of end stage liver disease
PEG-IFN: Pegylated interferon
PI: Protease inhibitor
PR: PEG-IFN plus RBV
RBV: Ribavirin
RNA: Ribonucleic acid
RVR: Rapid virological response
SVR: Sustained virological response
TVR: Telaprevir
TW: Treatment week
